# A Multiphysics Thermoelastoviscoplastic Damage Internal State Variable Constitutive Model including Magnetism

**DOI:** 10.3390/ma17102412

**Published:** 2024-05-17

**Authors:** M. Malki, M. F. Horstemeyer, H. E. Cho, L. A. Peterson, D. Dickel, L. Capolungo, M. I. Baskes

**Affiliations:** 1 Aerospace and Automotive Department, International University of Rabat, Rabat 11103, Morocco; mounia.malki@uir.ac.ma; 2 School of Engineering, Liberty University, Lynchburg, VA 24515, USA; 3 Center for Advanced Vehicular Systems, Mississippi State University, Starkville, MS 39759, USA; lukep@cavs.msstate.edu; 4 Department of Mechanical Engineering, Mississippi State University, Starkville, MS 39762, USA; doyl@me.msstate.edu; 5 Materials Science and Technology Division, Los Alamos National Laboratory, Los Alamos, NM 87545, USA; laurent@lanl.gov; 6 Department of Materials Science & Engineering, University of North Texas, Denton, TX 76203, USA; michael.baskes@unt.edu

**Keywords:** magnetism, magnetostrictive strain, magneto-mechanical effect, ferromagnets

## Abstract

We present a macroscale constitutive model that couples magnetism with thermal, elastic, plastic, and damage effects in an Internal State Variable (ISV) theory. Previous constitutive models did not include an interdependence between the internal magnetic (magnetostriction and magnetic flux) and mechanical fields. Although constitutive models explaining the mechanisms behind mechanical deformations caused by magnetization changes have been presented in the literature, they mainly focus on nanoscale structure–property relations. A fully coupled multiphysics macroscale ISV model presented herein admits lower length scale information from the nanoscale and microscale descriptions of the multiphysics behavior, thus capturing the effects of magnetic field forces with isotropic and anisotropic magnetization terms and moments under thermomechanical deformations. For the first time, this ISV modeling framework internally coheres to the kinematic, thermodynamic, and kinetic relationships of deformation using the evolving ISV histories. For the kinematics, a multiplicative decomposition of deformation gradient is employed including a magnetization term; hence, the Jacobian represents the conservation of mass and conservation of momentum including magnetism. The first and second laws of thermodynamics are used to constrain the appropriate constitutive relations through the Clausius–Duhem inequality. The kinetic framework employs a stress–strain relationship with a flow rule that couples the thermal, mechanical, and magnetic terms. Experimental data from the literature for three different materials (iron, nickel, and cobalt) are used to compare with the model’s results showing good correlations.

## 1. Introduction

In recent years, automotive electrification has served as an efficient technology to reduce fuel consumption, greenhouse gas emissions (GHG), and dependency on volatile resources, as well as maintain the high-power density and efficiency of a motor [1,2]. A new generation of electric propulsion motors is based on magnetic materials (iron (Fe), nickel (Ni), and cobalt (Co) for example), including soft magnetic laminations and Rare Earth (RE) elements (e.g., Neodymium or Cerium). Magnets exhibit a rich variety of material behavior originating from their type (diamagnet, paramagnet, ferromagnet, antiferromagnet, and ferrimagnet) and microstructural behavior (magnetic domains interaction and electron spin) that are strongly dependent on temperature, mechanical stress, external magnetic field, damage within the material, and time.

To meet the high automotive market requirements, the electric cars industry used Permanent Magnets (PM) and Rare-Earth (RE) magnets to increase the engine power at a low price. Permanent magnets are critical components for electric motors and power generators. However, RE elements are earth sources that decrease on a daily basis, thus requiring a high market value and becoming more expensive. According to the US Department of Energy (DOE) and other international institutes [3], RE elements are critical elements that are subjected to high supply risks, since the main location of RE elements is in China. To date, RE elements are non-recyclable elements; therefore, finding a cheap and more available alternative is an emerging issue to address. In an effort to fill this engineering gap, scientists dedicated considerable efforts to find an alternative to permanent magnets by understanding the physical behavior of magnets and modeling it in a mathematical framework that is used for various industrial applications. 

Previous models describe the mechanical response of a magnetic sample when subjected to an external magnetic field [4,5]. The mechanical response comes, in most cases, as a mechanical dimensional elongation [6]. This effect is called magnetostriction [6,7] and is mostly found in ferromagnetic materials and giant magnets [8]. Bozorth (1945) [9] and Brown (1949) [10] first presented a theoretical analysis of the magneto-mechanical effect in ferromagnetic materials by replacing the applied stress with an equivalent field. Afterward, Cullity (1972) [11] researched such problems using Le Chatelier’s principle. Sablik et al. (1988) [12] considered the changes in the hysteresis of ferromagnetic materials under constant stress. More systematic research on the magneto-mechanical effects was presented by Jiles (1995) [13] and Jiles and Atherton (1986) [14] based on the concepts of an “effective field theory” and “law of approach”.

Although various models have been previously introduced to solve such intricate engineering problems [15,16,17], most studies individually considered mechanical, magnetic effects, and thermal effects. None of the previously cited models coupled the mechanical, thermal, and damage effects with magnetic effects in a consistent model. 

Only a handful of studies on magnetoplasticity have been completed, and the history is fairly recent. Zagoruiko (1965) [18] was one of the first researchers to address the magneto-plasticity effect when he demonstrated that a pulsed magnetic field affected the plasticity of NaCl crystals. Later, Kravchenko (1970) [19] showed that the presence of a magnetic field inversely affects the metal’s plasticity. Later, Al’shits (1987) [20] showed that a static magnetic field can affect the plastic behavior of NaCl. Molotskii (2000) [21] showed that when magnetic field transitions between singlet and triplet states occur, the depinning of dislocations from obstacles is facilitated, thus increasing plasticity. Mullner et al. (2003) [22] showed how a magnetic field changed the stress–strain behavior of a single crystal Ni-Mn-Ga. An experimental study also showed that the yield strength can decrease by ~5%, but the ductility can increase by ~15% [23]. In other studies, however, the hardness of steel increased under the magnetic field [24,25], and some studies even showed that both ductility and tensile strength increased [26,27]. Many laboratory experiments have shown that the mechanical properties of materials can be affected by precipitation and phase transformation kinetics altered by the applied magnetic field [28,29]. For instance, AISI 8620 steel showed some changes in the amount of cementite and its distributions with exposure to a magnetic field [28]. In their study, the cementite increased with the magnetic field while temperature was maintained low, which implies the altered precipitation is due to the magnetic field instead of temperature. Murase et al. (1993) [30] also showed that the fracture toughness of austenitic steels decreased by approximately 20% at a magnetic strength of 8 T. This embrittlement was due to the martensitic transformation induced by the magnetic field as it decreases austenite stability. Other than the effects on phase transformation and precipitation, several studies reported that magnetic fields influence fracture toughness [31,32,33], ductility [24,28,32,34,35], fatigue life [23,25,28,36,37,38,39,40,41], and creep [42] through various and complex mechanisms. Interestingly, Mullner et al. (2003) [22] reported that the crystallographic orientations (texture) and twins under a magnetic field change the magnetization across the twins and thus affect the dislocations in polycrystalline Ni-Mn-Ga. A useful review summarizing recent research progress regarding microstructure and property of metals influenced by magnetic field can be also found in Hu et al. (2022) [43]. 

Several studies have been conducted to develop constitutive models to account for the magnetostriction and magnetoplasticity behavior of magnetic materials. In recent studies, Zheng and Liu (2005) [44] proposed a constitutive relation for largely (non-linear) magnetostrictive straining, particularly for Terfenol-D rods. Li and Xu (2011) [45] modified the classical model by Jiles and Atherton (1984) [46] and Sablik (1997) [47] to account for asymmetry in magneto-mechanical behavior arising from different loading directions (tensile and compressive). They incorporated a stress demagnetization effect, a variable domain pinning coefficient, stress-dependent saturation magnetostriction, and a domain coefficient dependent on applied stress. Wang et al. (2011) [48] integrated the plastic deformation effect into a magneto-mechanical constitutive model. More recently, Daniel (2018) [49] presented a useful analytical model with only three parameters that captures stress-dependent magnetostriction deformation based on the energy consideration. Shi et al. (2020) [50] proposed a magnetoelastoplastic model from the standpoint of magnetic memory, and Shi (2020) [51] also presented a magnetoelastoplastic constitutive relation by using magnetoelastic energy and magnetoplastic energy due to domain pinning. In this historical context, a theoretical study on the thermomechanical effects coupled with damage and magnetic effects is of great interest to provide a physical basis for various magnetic testing techniques and gives a better understanding of the test results. Therefore, a comprehensive model framework for predicting magnetically influenced deformation behavior is needed. In the present study, we report a magnetism-dependent elastoviscosplastic and damage model in the framework of Internal State Variables constitutive theory, which incorporates aspects of kinematics, thermodynamics, and kinetics of deforming continuum body under magnetic, thermal, and mechanical loads.

The Internal State Variable (ISV) theory has been viable over the past years starting from the significant contribution of Onsager (1931) [52] related to thermodynamics. Then, Eckart (1948) [53] used ISV theory in continuum mechanics. Kroner (1960) [54] postulated that the ISV continuum theory can use physically observed behaviors although the complete microstructure arrangement is unnecessary as long as the macroscale ISV representation is complete. Later, Coleman and Gurtin (1967) [55] proposed the use of history-dependent variables to quantify dissipative mechanisms of internal deformation within a thermodynamically consistent framework. Hence, an ISV model employs a set of constitutive equations that capture the history effects of a material to predict its mechanical properties and its future behavior based on the already existing mathematical state description [56]. Many models are based on Coleman and Gurtin’s (1967) [55] framework, and they are summarized by Horstemeyer and Bammann’s (2010) [57] historical review of ISV theory. 

The deformation gradient maps the deformation from the reference configuration to the current configuration. The multiplicative decomposition of the deformation gradient describes the deformation of elastic–thermal–magnetic behavior. Previous researchers used kinematic decomposition [58,59,60,61,62,63,64,65,66,67,68,69,70,71,72,73]; to establish a constitutive model for deformations of different materials (such as thermal effects [71]). Later, Dimitrov et al. (2020) [74] extended the thermomechanical description to electrothermomechanical constitutive equations to relate the electric effects on the thermomechanical hardening of the metals. Recently, Cho et al. (2022) [75] incorporated the nuclear irradiation effect on the elastoviscoplastic behavior of crystalline metals but not for magnetic effects heretofore.

The contribution of our work includes the development of an Internal State Variable (ISV) constitutive model that accounts for magnetism-dependent elasto-viscoplasticity and damage for magnetic materials that brings in three novel ideas: (1) introduction of kinematics for the deforming continuum body under an external magnetic field to account for elastic/inelastic deformation and vorticity affected by the magnetic field; (2) introduction of a new magnetic ISV constrained by the first and second laws of thermodynamics (Clausius–Duhem inequality); and (3) this ISV-based constitutive model is a novel approach to address the kinetics of mechanical, thermal, and magnetic boundary conditions. The kinematics, thermodynamics, and kinetics of an ISV model should be independently developed but internally consistent, and we generate the theory for magnetic-influenced deformation herein. The contribution of this work is twofold. First, the physical establishment of how magnetic effects, resulting from a material’s proper magnetization or a material subjected to an externally applied field, can change the behavior of a structure on the macroscale level. Second, the development of a consistent thermodynamic model following the Coleman and Gurtin (1967) [55] thermodynamic framework satisfies the first and second laws of thermodynamics. The first section of this document presents an introduction to the problem statement. Section 2 presents the macroscale deformation behavior exhibiting a response to an external magnetic field admitting subscale information from the mesoscale and the nanoscale. Section 3 provides a full description of the model’s kinematics relations of the thermal–elastic–damage–magnetic ISV model. The Coleman and Gurtin (1967) [55] thermodynamic framework of the ISV model is presented in Section 4. To describe the macroscale behavior of the material, the kinetics of the ISV model are presented in Section 5. Finally, we present model comparison with lab experimental data for magnetostriction and magnetization behavior. 

A standard notation is followed in this mathematical formulation. In this text, tensors are denoted by **boldface** font while scalar values have the standard weight. All tensor components are written with respect to a fixed Cartesian coordinate system. Special care is given to specify configurations throughout the derivation by using accent marks where the tilde ($\overset{ˇ}{R}$), circumflex ($\hat{R}$), macron ($\bar{R}$), double macron ($\overset{̿}{R}$), and overbrace ($\overbrace{R}$) represent different intermediate configurations. The following definitions are used in the text: $AB=>{\left(A.B\right)}_{ij}=A_{ik}B_{kj},A;B=A_{ij}B_{ij},\;tr\left(A\right)=A_{ii},{\left(A^{T}\right)}_{ij}=A_{ji}$. The overdot denotes the material time derivative. The apostrophe denotes the co-rotational derivative.

## 2. Phenomenological Behavior

In nature, several types of magnetic materials exist. Differences in magnet types depend on material microstructure properties and response to external magnetic fields. The different types of magnets are diamagnets, paramagnets, ferromagnets, antiferromagnets, and ferrimagnets, and these are summarized in Table 1. Brugmans (1778) [76] characterized diamagnetism as the tendency of the material to oppose an applied magnetic field (*H*). Diamagnetism creates a repulsive force, and paramagnetism creates an attractive force when subjected to an applied magnetic field (*H*), making the total magnetic field stronger [77]. The third type of magnetism is ferromagnetism. Ferromagnetism is characterized by a spontaneous and strong magnetic field without the presence of an externally applied field. The three main ferromagnetic elements that exist in nature are the following: iron (Fe), nickel (Ni), and cobalt (Co), which are used to demonstrate our theory. Antiferromagnetism tends to have electrons with intrinsic magnetic moments that do not align parallel with each other but align in antiparallel orientations [78]. The fifth type of magnetism is ferrimagnetism. A ferrimagnet has the same properties as a ferromagnet, such that it retains a magnetic field even when no external magnetic field is applied but has a net magnetization less than that of ferromagnets alone.

The magnet types exhibit behavior that extends to multiscale levels. The effects of magnetism are described at the macroscale, mesoscale, and nanoscale as presented in the following section.

### 2.1. Macroscale Level: The Magnetostriction Phenomenon

Magnetostriction is a phenomenon found in ferromagnetic materials [7,15,79,80]. The magnetostriction phenomenon arises from the misalignment of magnetic domains such that, when subjected to an externally applied magnetic field, the domains align parallel to the magnetic field direction, resulting in a shape change at the macroscale level. Joule (1842) was the first to identify magnetostriction by observing a sample of nickel expanding when subjected to an external magnetic field. The concept of magnetostriction is a key feature employed in the magneto-mechanical coupled constitutive model described herein. 

Magnetostriction phenomena involve elastic, magnetic, and thermal effects. Magnetostriction is of great industrial interest for use in sensors, actuators, adaptive structures, robotics, and transducers [81]. They are widely used in the field of nondestructive evaluation [82]. The essence of magnetostriction is the dependence of mechanical strain on magnetization. MagnetoStrictive Materials (MSMs) are a class of smart materials that transfer energy from one form to another form; for example, they can convert magnetic energy into mechanical energy (Joule effect [83,84]) and vice versa (Villari effect, c.f. [84]). 

**Figure 1:**
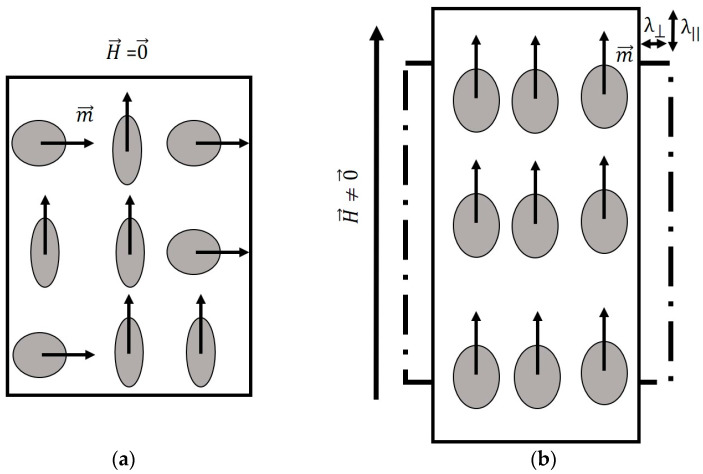
A nine-atom lattice showing the effect of an external magnetic field ($\vec{H}$) on the atom for which $\vec{m}$ is the magnetic moment. (**a**) Magnetic moments when no external magnetic field is applied, and (**b**) magnetic moments when subjected to a vertical external magnetic field. Two strain components appear: a parallel strain ($\lambda_{ǁ}$) and a perpendicular one ($\lambda_{⟘}$).

MSMs can exhibit large mechanical deformations in different directions when subjected to a strong external magnetic field. This behavior is due to the rotations of small magnetic domains (that exist inside of the grain) within the material, which are arbitrarily oriented when the material is not subjected to an external magnetic field. The orientations of these small domains change by the imposition of the magnetic field. The domain moments align themselves parallel to the externally applied field direction, thus creating a strain field, resulting in a noticeable mechanical elongation. As the intensity of the magnetic field increases, the magnetic domains tend to orient themselves in order to co-align their principal axes with the magnetic field in each region until saturation is reached. This effect is described in a small crystalline sample of a ferromagnetic material as illustrated in Figure 1.

**Figure 2:**
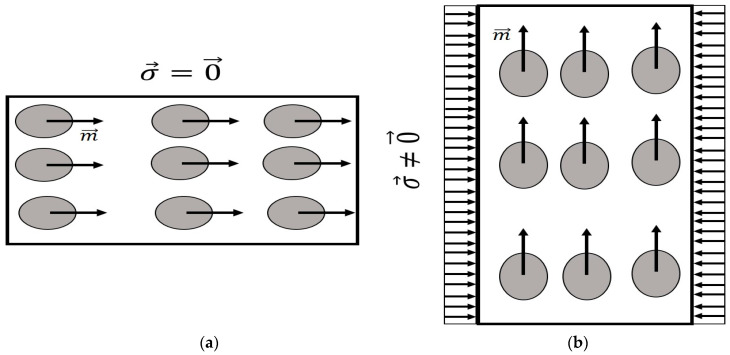
A nine-atom lattice showing the effect of a compressive uniformly distributed stress ($\vec{\sigma }$) on the magnetic properties of the atoms presented as the magnetic moment ($\vec{m}$). (**a**) Non-presence of stress illustrates a horizontal orientation of the magnetic moment. (**b**) The presence of compressive stress ($\vec{\sigma }$) results in a direction change in the magnetic moment ($\vec{m}$) (magnetic moments pointing up).

Bieńkowski and Kulikowski (1980) and Jiles (1995) demonstrated the existence of a mechanism reciprocal to magnetostriction [13,85]. The mechanism, called the Villari effect, involves a change in magnetization induced by mechanical stress. The Joule and Villari effects are observed in ferromagnets, antiferromagnets, and ferrimagnetic objects. Figure 2 illustrates the Villari effect for a crystal lattice structure. When the lattice is subjected to a mechanical stress parallel to the original magnetic moments, the magnetization of the sample rotates. Note that up to this point, the previously described effects were limited to temperatures lower than the Curie temperature, which is the temperature above which the material loses its magnetic properties [86,87]. Once the temperature of these materials exceeds the Curie temperature, the magnetic properties of the material are lost.

### 2.2. Mesoscale Level: Domain Wall Motion

**Figure 3:**
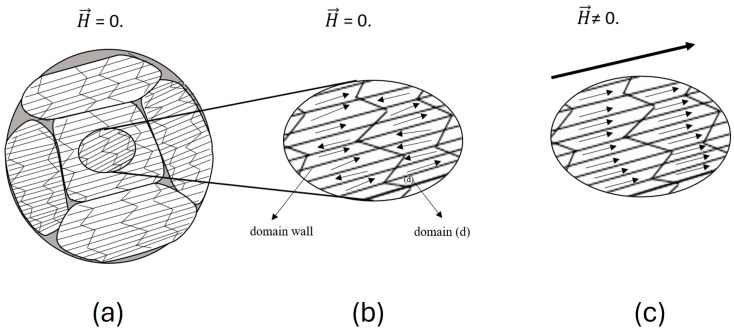
(**a**) Polycrystalline structure showing the magnetic domains and their appropriate magnetization ($\vec{m}$) direction when no external magnetic field ($\vec{H}$) is applied. (**b**) Magnified region of the polycrystalline structure, with no external magnetic field applied, and (**c**) magnified region when an external magnetic field ($\vec{H}$) is applied. The magnetic domain’s direction aligns with the external magnetic field direction.

In most cases, magnetostrictive strains exhibit a nonlinear behavior with respect to the external magnetic field. The nonlinear behavior is due to the domain structure within the grains and the grain orientation of the microstructure under study. Magnetic domains are the heart of magnetic material deformation. Figure 3 shows the domain structure of a magnetostrictive alloy. In Figure 3, the neighboring domains tend to have different magnetic moment orientations. The different alignment minimizes the magnetic energy within the specimen. Each domain (d) has a magnetization that can be expressed by [11]

(1) $${\vec{M}}_{d}=M_{s}\vec{\gamma },$$

such that ${\vec{M}}_{d}$ is the magnetization of the domain, $\vec{\gamma }$ represents the vector orientation of the axis on which most of the material’s magnetization is fixed, and $M_{s}$ represents the saturation magnetization value of each domain.

The moments of magnetic domains tend to rotate when exposed to a magnetic field until the magnetic domains’ direction is aligned with the magnetic field direction [88]. Thus, the domain walls, which are considered the transition region between the domains, start to move and elongate due to domain wall motion [14]. Domains whose orientation is closer to the magnetic field direction tend to elongate through the process while the others tend to shrink. Domain elongation and shrinkage result in a dimensionless change on the macroscale level (the Joule effect). Domain growth stops once saturation magnetization is reached.

### 2.3. Nanoscale Level: Ising Model

At the nanoscale level, electron spins play an important role in moving domain walls. This physical behavior is explained through the Ising model [89,90,91]. The Ising model is a statistical model used to describe ferromagnetic behavior in terms of phase transitions and the magnetic domain motion. The Ising model was initially developed to solve a one-dimensional problem under the assumption of no phase transitions. The model is based on defining two spinning variables that represent the magnetic dipole moments of the atomic spins. For a two-dimensional (2D) lattice, each lattice site has a local magnetic moment and is represented by an arrow pointing up (for a positive magnetic moment) and an arrow pointing down (for a negative magnetic moment). The moment is assumed to be equal to +1 when the spins are pointing up or to −1 when the spins are pointing down. The Ising model is used to compute the magnetization order (O) using

(2) $$O=\left\langle \frac{K_{+}-K_{-}}{K}\right\rangle ,$$

such that *K* represents the total number of spins in the lattice, $K_{+}$ is the number of positive spins, and $K_{-}$ is the number of negative spins. The magnetization order in Equation (2) represents the expectation value of the magnetic moment ($\mu \left(K_{+}-K_{-}\right)$) relative to the largest possible magnetic moment (μK) such that μ is the magnetic moment. 

In terms of energy, the Ising model [89] includes two contributions: the first characterizes how neighboring spins affect the spin, and the second contribution characterizes how an applied magnetic field affects each spin within the lattice. This statement is written in the following way:

(3) $$E=-J\sum_{\left\langle i,j\right\rangle }g_{i}g_{j}-H\sum_{i}g_{i},$$

such that *E* is the total energy, *J* is the positive coefficient giving the interaction strength, and $g_{i}$ is the spin variable corresponding to direction values (=+1 or −1). The first term of Equation (3) represents the neighboring spin’s interaction, while the second term represents the effect of the applied field on each spin.

## 3. Kinematics

In continuum mechanics, a three-dimensional material subjected to a magneto-thermo-mechanical deformation can be described using the deformation gradient concept to map a deformation from the reference (initial) configuration (*R*_0_) to the current configuration (*R*) with possible intermediate configurations in between. The deformation gradient mapping a particle from its initial position X to the current position ***x*** is given as follows [58,92]:

(4) $$F=\frac{\partial x}{\partial X},$$

such that X is the displacement in the reference configuration (*R*_0_) and x is the displacement in the current configuration (*R*). The deformation gradient assumes continuity, where the local deformation at X is characterized as the gradient of the motion, which is a second-order two-point tensor. 

For the continuum model herein, we need to define the Eulerian and the Lagrangian strains in a classical manner [93]. The Lagrangian finite strain tensor with respect to the reference configuration is defined as follows:

(5) $$E=\frac{1}{2}\left(F^{T}F-I\right),$$

with I as the identity matrix.

**Figure 4:**
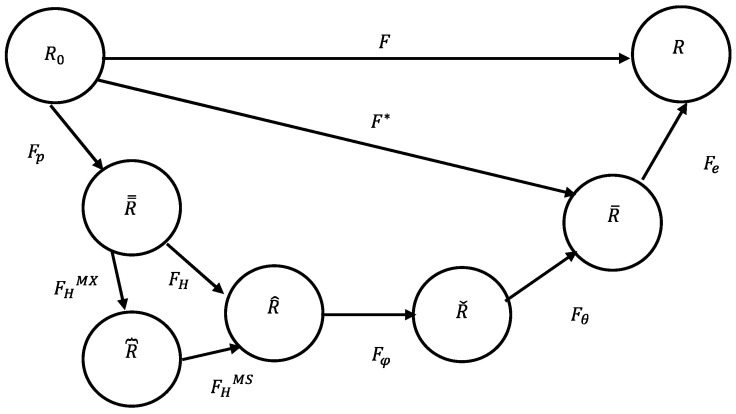
Multiplicative decomposition of the deformation gradient into the plastic (*p*), magnetic (*H*), damage (φ), thermal (*θ*), and elastic parts (*e*).

For large strains, a multiplicative decomposition of the deformation gradient [93] into plastic, damage, magnetic, thermal, and elastic parts is performed as schematically illustrated in Figure 4. The total deformation gradient is therefore written as

(6) $$F=F_{e}F_{\theta }F_{\varphi }F_{H}F_{p},$$

where the total deformation gradient can be multiplicatively decomposed into elastic ($F_{e}$), thermal ($F_{\theta }$), damage ($F_{\varphi }$), magnetostrictive ($F_{H}$), and plastic ($F_{p}$) deformation gradients. Note that the thermomechanics in our constitutive model represents the thermal contribution to the deformation. For instance, our model takes the thermal contribution into consideration of the kinematics to track the elastic and inelastic deformation of the material as shown in Equation (6) and Figure 4.

The magnetic deformation gradient ($F_{H}$) is multiplicatively decomposed into two sub-deformation gradients in this model:

(7) $$F_{H}=F_{H}^{MS}F_{H}^{MX},$$

where the first sub-deformation gradient ($F_{H}^{MS}$) is related to the magnetostriction elongation effect and the second sub-deformation gradient ($F_{H}^{MX}$) is related to the Maxwell magnetic field effects created by the externally applied field (***H***). Generally, the Maxwell field effects on the deformation of the material are so small that they are not taken into consideration in previously developed models. However, the purpose of this model is to provide a full description of the magnetic material’s behavior; therefore, all the effects are included. The total deformation gradient (in Equation (6)) can be simplified to a product of inelastic $\left(F_{*}\right)$ and elastic ($F_{e}$) deformation gradient components,

(8) $$F=F_{e}F_{*},$$

such that $F_{*}$ represents all the inelastic deformations $F_{*}=F_{\theta }F_{\varphi }F_{H}F_{p}$.

The first intermediate configuration ($\overset{̿}{R}$) is defined by the plastic deformation gradient ($F_{p}$). The second intermediate configuration ($\hat{R}$) is defined by the multiplication of the magnetic deformation gradient ($F_{H}$) and the plastic deformation gradient ($F_{p}$): $F_{H}F_{p}$. 

The third intermediate configuration ($\overset{ˇ}{R}$) is defined by the multiplication of the damage deformation gradient ($F_{\varphi }$), the magnetic deformation gradient ($F_{H}$), and the plastic deformation gradient ($F_{p}$): $F_{\varphi }\;F_{H}F_{p}.$ The magnetic deformation gradient also depends on the damage since the presence of voids/cracks may modify the motion of the domain walls, known as the domain wall pinning effect [94]. Domain wall pinning can arrest material elongation caused by an external magnetic field. Moreover, a high number of heterogeneities (particles, voids) leads to a decrease in permeability (μ) and an increase in coercivity ($H_{c}$) [95]. 

The fourth intermediate configuration ($\bar{R}$) is defined by the multiplication of the thermal deformation gradient ($F_{\theta }$), the magnetic deformation gradient ($F_{H}$), the damage deformation gradient ($F_{\varphi }$), and the plastic deformation gradient ($F_{p}$):$F^{*}$, such that ${F^{*}=F}_{\theta }F_{\varphi }{F_{H}F}_{p}.$ Both magnetic and damage behavior characteristics of a material are sensitive to temperature. A permanent magnet can lose its properties once a critical temperature (Curie temperature) is reached. Damage mechanisms and evolutionary rates vary with temperature. The elastic deformation gradient serves to describe unloading elastically through $F_{e}^{-1}$. The thermal deformation gradient and damage deformation follow Francis et al. (2014) [71]. Finally, the plastic deformation gradient is the last one since the inelastic flow rule is a function of thermal and damage effects. 

For our interest, the constitutive equations are developed in intermediate configuration $\hat{R}$, where all magnetic deformations happen. The deformation gradient tensors in their corresponding intermediate configurations are mathematically defined as follows:

(9) $$F^{*}=\frac{\partial \bar{x}}{\partial X},F_{p}=\frac{\partial \overset{̿}{x}}{\partial X},F_{H}=\frac{\partial \hat{x}}{\partial \overset{̿}{X}},F_{\varphi }=\frac{\partial \tilde{x}}{\partial \hat{X}},\;F_{\theta }=\frac{\partial \bar{x}}{\partial \hat{X}},F_{e}=\frac{\partial x}{\partial \bar{X}}.$$

The Jacobian of the total deformation gradient, which is the change in volume between the reference (*R*_0_) and current (*R*) configurations, is given as

(10) $$J=\det\left(F\right)=\det\left(F_{p}\right)\det\left(F_{H}\right)\det\left(F_{\varphi }\right)\det\left(F_{\theta }\right)\det\left(F_{e}\right),$$

such that the Jacobian of each deformation gradient represents the conservation of the mass of the system, given as follows:

(11) $$\det\left(F_{p}\right)=J_{p}=\frac{\overset{̿}{V}}{V_{0}},\det\left(F_{H}\right)=J_{H}=\frac{\hat{V}}{\overset{̿}{V}},\det\left({F_{H}}^{MX}\right)={J_{H}}^{MX}=\frac{\overbrace{V}}{\overset{̿}{V}},\det\left({F_{H}}^{MS}\right)={J_{H}}^{MS}=\frac{\hat{V}}{\overbrace{V}},\det\left(F_{\varphi }\right)=J_{\varphi }=\frac{\tilde{V}}{\hat{V}},\;\det\left(F_{\theta }\right)=J_{\theta }=\frac{\bar{V}}{\overset{ˇ}{V}},\;\mathrm{and}\;\det\left(F_{e}\right)=J_{e}=\frac{V}{\bar{V}}.$$

Based on previous work by Bammann and Aifantis (1989) [61], the damage deformation gradient is expressed as follows:

(12) $$F_{\varphi }=\frac{1}{{\left(1-\varphi \right)}^{\frac{1}{3}}}I.$$

The Jacobian of the damage deformation gradient is the following [96]:

(13) $$\det\left(F_{\varphi }\right)=\frac{1}{\left(1-\varphi \right)}.$$

Similarly, Bammann and Solanki (2010) [97] defined the Jacobian of the thermal deformation gradient as follows:

(14) $$\det\left(F_{\theta }\right)={F_{\theta }}^{3}.$$

The developed model assumes a linear thermal expansion that can be assumed for the thermal deformation gradient tensor ($F_{\theta }$) and is given as

(15) $$F_{\theta }=\left(1+\alpha_{th}∆\theta \right)I,$$

where $\alpha_{th}$ is the thermal expansion coefficient and θ is the temperature.

Assuming deviatoric plastic deformation, the Jacobian of the plastic deformation gradient is unity,

(16) $$\det\left(F_{p}\right)=1.$$

From the total deformation gradient, the total Lagrangian strain tensor is obtained using additive decomposition in the reference configuration

(17) $$E=E_{e}+E_{\theta }+E_{\varphi }+E_{H}+E_{p},$$

where

(18) $$\begin{array}{c} E=\frac{1}{2}\left(C-I\right),{\bar{E}}_{e}=\frac{1}{2}\left({\bar{C}}_{e}-I\right),{\overset{ˇ}{E}}_{\theta }=\frac{1}{2}\left({\overset{ˇ}{C}}_{\theta }-I\right),{\hat{E}}_{\varphi }=\frac{1}{2}\left({\hat{C}}_{\varphi }-I\right),{\overset{̿}{E}}_{H}=\frac{1}{2}\left({\overset{̿}{C}}_{H}-I\right),\\ {\overbrace{E}}_{H}^{MS}=\frac{1}{2}\left({\overbrace{C}}_{H}^{MS}-I\right),\;{\overset{̿}{E}}_{H}^{MX}=\frac{1}{2}\left({\overset{̿}{C}}_{H}^{MX}-I\right),\;\mathrm{and}E_{p}=\frac{1}{2}\left(C_{p}-I\right), \end{array}$$

and ***C*** is the Cauchy–Green deformation tensor. Pulling back all the intermediate Lagrangian tensors to the reference configuration, we obtain the following:

(19) $$\begin{array}{l} E_{e}=F_{p}^{T}F_{H}^{T}F_{\varphi }^{T}F_{\theta }^{T}{\bar{E}}_{e}F_{\theta }{F_{\varphi }F}_{H}F_{p},\\ E_{\theta }=F_{p}^{T}F_{H}^{T}F_{\varphi }^{T}{\overset{ˇ}{E}}_{\theta }{F_{\varphi }F}_{H}F_{p},\\ E_{\varphi }=F_{p}^{T}F_{H}^{T}{\hat{E}}_{\varphi }F_{H}F_{p},\\ E_{H}=F_{p}^{T}{\overset{̿}{E}}_{H}F_{p}. \end{array}$$

The stretch tensors of each Lagrangian tensor are a strain measure in terms of material coordinates and can be obtained when the deformation gradients are determined as follows:

(20) $$C=F^{T}F,{\bar{C}}_{e}=F_{e}^{T}F_{e},{\overset{ˇ}{C}}_{\theta }=F_{\theta }^{T}F_{\theta },{\hat{C}}_{\varphi }=F_{\varphi }^{T}F_{\varphi },\;{\overset{̿}{C}}_{H}=F_{H}^{T}F_{H},\;{\overset{̿}{C}}_{H}^{MX}={\left(F_{H}^{MX}\right)}^{T}F_{H}^{MX},\;{\overbrace{C}}_{H}^{MS}={\left(F_{H}^{MS}\right)}^{T}F_{H}^{MS},\;\mathrm{and}\;C_{p}=F_{p}^{T}F_{p}.$$

Each Cauchy–Green deformation tensor (***C***) may be subjected to spectral decomposition of the form

(21) $$C=\sum_{i=1}^{3}\lambda_{i}^{2}n_{i}⊗n_{i},$$

where the stretch ratio, $\lambda_{i}$, is the square root of each positive eigenvalue that corresponds to each orthonormal vector, $n_{i}$. Each deformation gradient tensor has a polar decomposition of the form

(22) $$F_{•}=R_{•}U_{•},$$

where (•) can be any of the terms resulting from the deformation gradient decomposition (*p*, H, φ, θ, *e*). The relationship between ***C*** and ***U*** is

(23) $$U=\sqrt{C}=\sum_{i=1}^{3}\lambda_{i}n_{i}⊗n_{i},$$

where the directions (eigenvectors) ($n_{i}$) remain unchanged, and the principal stretch ratios (*λ_i_*) are used. 

The scalar form of the damage right stretch tensor that affects the damage internal state variables, defined by Bammann and Solanki (2010) [97], is defined in the damage-associated configuration ($\hat{R}$) as follows:

(24) $${\hat{t}}_{\varphi }=\frac{1}{3}tr\left({\hat{C}}_{\varphi }\right)=\frac{1}{{\left(1-\varphi \right)}^{\frac{1}{3}}}I,$$

for which the corresponding time derivative is given as follows:

(25) $${\dot{\hat{t}}}_{\varphi }=\frac{\dot{\varphi }}{{3*\left(1-\varphi \right)}^{\frac{4}{3}}}I=\frac{\dot{\varphi }}{3*\left(1-\varphi \right)}{\hat{t}}_{\varphi }I=\frac{1}{3}I:{\dot{\overset{ˇ}{C}}}_{\varphi }.$$

The velocity gradient associated with the deformation of the current configuration is decomposed into elastic, thermal, magnetic, damage, and plastic components:

(26) $$l=\dot{F}F^{-1}=l_{e}+l_{*}=l_{e}+l_{\theta }+l_{\varphi }+l_{H}+l_{p}=l_{e}+l_{\theta }+l_{\varphi }+{l_{H}}^{MS}+{l_{H}}^{MX}+l_{p},$$

where ($l_{e}$) is the elastic velocity gradient, ($l_{\theta }$) is the thermal velocity gradient, ($l_{H}$) is the magnetic velocity gradient, ($l_{\varphi }$) is the damage velocity gradient, and ($l_{p}$) is the plastic velocity gradient. Each velocity gradient can be written in terms of the deformation gradients as follows:

(27) $$l_{e}={\dot{F}}_{e}F_{e}^{-1},{{l_{\theta }=F_{e}{\dot{F}}_{\theta }F_{\theta }^{-1}F_{e}^{-1},l}_{\varphi }=F_{e}F_{\theta }{\dot{F}}_{\varphi }F_{\varphi }^{-1}F_{\theta }^{-1}F_{e}^{-1},l}_{H}=\;F_{e}F_{\theta }F_{\varphi }{\dot{F}}_{H}F_{H}^{-1}F_{\varphi }^{-1}F_{\theta }^{-1}F_{e}^{-1},\;\mathrm{and}\;l_{p}=F_{e}F_{\theta }F_{\varphi }F_{H}{\dot{F}}_{p}F_{p}^{-1}F_{H}^{-1}F_{\varphi }^{-1}F_{\theta }^{-1}F_{e}^{-1}.$$

The velocity gradients in the intermediate $\hat{R}$ configuration is obtained by pulling back the elastic, thermal, and damage velocity gradients ($F_{e}$), ($F_{\theta }$), and ($F_{\varphi }$) and pushing forward the plastic velocity gradient ($F_{p}$). This results in the following velocity gradients:

(28) $$\begin{array}{l} {\hat{l}}_{e}=F_{\varphi }^{-1}F_{\theta }^{-1}F_{e}^{-1}{\dot{F}}_{e}F_{e}^{-1}F_{e}F_{\theta }F_{\varphi },\\ {\hat{l}}_{\theta }=F_{\varphi }^{-1}F_{\theta }^{-1}{\dot{F}}_{\theta }F_{\theta }^{-1}F_{\theta }F_{\varphi }=F_{\varphi }^{-1}F_{\theta }^{-1}{\dot{F}}_{\theta }F_{\varphi },\\ {\hat{l}}_{\varphi }=F_{H}^{-1}{\dot{F}}_{\varphi }F_{\varphi }^{-1}F_{H},\\ {\hat{l}}_{H}={\dot{F}}_{H}F_{H}^{-1},\\ {\hat{l}}_{p}={F_{H}\dot{F}}_{p}F_{p}^{-1}F_{H}^{-1}. \end{array}$$

Velocity gradient l can be decomposed into two parts, the skew and symmetric parts:

(29) l=D+W,

where ***D*** is the symmetric rate of deformation tensor and ***W*** is the asymmetric spin tensor:

(30) $$D=sym\left(l\right)=\frac{1}{2}\left(l+l^{T}\right),\mathrm{and}W=skew\left(l\right)=\frac{1}{2}\left(l-l^{T}\right).$$

The total rate of deformation is additively decomposed into elastic, plastic, damage, magnetic, and thermal deformation rates by additive decomposition as follows:

(31) $$D=D_{e}+D_{\theta }+D_{\varphi }+D_{H}+D_{p},$$

where ***D_e_***, ***D_θ_***, $D_{\varphi }$, ***D_H_***, and ***D_p_*** are the elastic, thermal, damage, magnetic, and plastic components of the rate of deformation. Likewise, the spin tensor is additively decomposed as follows:

(32) $$W=W_{e}+W_{\theta }+W_{\varphi }+W_{H}+W_{p},$$

where the thermal spin and the damage spin are assumed to be equal to zero because the nondiagonal components of the velocity gradient are zero. Therefore, the total spin is written as follows:

(33) $$W=W_{e}+W_{H}+W_{P}.$$

The magnetic moment spin in this case refers to the spin moment resulting from the electron’s intrinsic motion. The spin moment resulting from other subatomic elementary particles (such as quarks in the protons and neutrons of the atomic nuclei) is assumed to be neglected because of its small magnetic moment. The magnetic spin influences the ordering of the electrons, nuclei in atoms, and molecules. A change in the ordering of the molecules induces a change in the magnetic domain orientation, resulting in a dimensional change appearing on the macroscale level of the material. The spin of a complete body is the sum of the spins of the elementary particles (electrons, neutrons, and protons),

(34) $$W_{H}=1/g[\chi D_{H}-D_{H}\chi ],$$

where g is the orientation spin variable arising from the Ising model and χ and ***D_H_*** are the kinematic magnetization term and the magnetic deformation rate tensor that are described in detail in the kinetics part of the model. This form is similar to the plastic spin [98]. Dafalias (1989) [98] showed that the plastic spin represents the rotation rate of the material with respect to its substructure during inelastic deformations. This physical behavior is expressed in terms of an equation relating the plastic spin to the plastic deformation rate tensor.

(35) $$W_{p}=-1/\varsigma [\beta D_{p}-D_{p}\beta ],$$

where *ζ* is the orientation coefficient and β is the kinematic hardening variable [98,99].

The strain rate is therefore given as follows:

(36) $$\dot{\varepsilon }={\dot{\varepsilon }}_{e}+{\dot{\varepsilon }}_{\theta }+{\dot{\varepsilon }}_{\varphi }+{\dot{\varepsilon }}_{H}+{\dot{\varepsilon }}_{p}.$$

The Cauchy stress (σ) is expressed as follows:

(37) $$\sigma =J_{e}^{-1}\tau =J_{e}^{-1}F_{e}\hat{S}F_{e}^{T},$$

where the Cauchy stress tensor (***σ***) and the first Piola Kirchhoff stress tensor (τ) are found in the current configuration, *R*, and the second Piola–Kirchhoff stress ($\hat{S}$) invoked the intermediate configuration, $\hat{R}.$

## 4. Thermodynamics

In this section, a thermodynamic model with internal state variables is developed to capture the path-dependent inelastic deformation processes in the intermediate configuration ($\hat{R}$) (where all magnetic deformations occur) and then mapped to the current configuration (*R*) [100].

The law of conservation of energy dictates that the rate of change in the internal energy of any Representative Volume Element (RVE) is equal to the rate of mechanical work of the net external force acting on that volume plus all other energies (magnetic energy in this model) that enter or leave the RVE. In local form, the first law of thermodynamics is given as follows:

(38) $$\rho \dot{u}=S:\dot{E}+\left(B.\dot{H}+\dot{B}.H\right)+\rho r-\nabla .q,$$

such that *u* is the specific internal energy, S is the Piola–Kirchhoff stress tensor, ***H*** = ***H***(***B***, ***M***) is the external magnetic field, ***B*** is the magnetic flux density, $\dot{H}$ is the external magnetic field rate, $\dot{B}$ is the magnetic flux density rate, *r* is the specific heat generation rate, ***q*** is the heat flux vector, and ρ is the density. Term $\left(B.\dot{H}+\dot{B}.H\right)$ includes the magnetoelastic and the Zeeman energies [101]. The magnetoelastic energy results from magnetostriction, while the Zeeman energy represents the interaction of the magnetic material and the externally applied magnetic field. 

In the intermediate configuration ($\hat{R}$), the first law of thermodynamics is written as follows [71,102]:

(39) $$\hat{\rho }\dot{\hat{u}}=\hat{S}:\dot{\hat{E}}+\left(\hat{B}.\dot{\hat{H}}+\dot{\hat{B}}.\hat{H}\right)+\hat{\rho }\hat{r}-\hat{\nabla }.\hat{q}.$$

The Clausius–Duhem (CD) inequality is given in the local form as follows [71,103]:

(40) $$\rho \dot{s}-\frac{1}{\theta }\rho r+\frac{1}{\theta }\nabla .q-\frac{1}{\theta^{2}}q.\nabla \theta \geq 0,$$

where *s* is the entropy of the material.

In the intermediate configuration ($\hat{R}$), the CD inequality is given as follows:

(41) $$\hat{\rho }\dot{\hat{s}}-\frac{1}{\theta }\hat{\rho }\hat{r}+\frac{1}{\theta }\hat{\nabla }.\hat{q}-\frac{1}{\theta^{2}}\hat{q}.\hat{\nabla }\hat{\theta }\geq 0.$$

The Helmholtz free energy in the intermediate configuration $\left(\hat{R}\right)$ is defined using the formulation of Coleman and Gurtin (1967) [55] as follow:

(42) $$\hat{\psi }=\hat{u}-\theta \hat{s},$$

and its time derivative is defined as follow:

(43) $$\dot{\hat{\psi }}=\dot{\hat{u}}-\dot{\theta }\hat{s}-\theta \dot{\hat{s}}.$$

Substituting Equation (43) into the energy balance relation in Equation (39) yields

(44) $$\hat{\rho }\left(\dot{\hat{\psi }}+\dot{\theta }\hat{s}+\theta \dot{\hat{s}}\right)=\hat{S}:\dot{\hat{E}}+\left(\hat{B}.\dot{\hat{H}}+\dot{\hat{B}}.\hat{H}\right)+\hat{\rho }\hat{r}-\hat{\nabla }.\hat{q}.$$

Substituting Equation (44) into the Clausius–Duhem inequality (Equation (41)) produces inequality

(45) $$-\hat{\rho }\dot{\hat{\psi }}-\hat{\rho }\dot{\theta }\hat{s}+\hat{S}:\dot{\hat{E}}+\left(\hat{B}.\dot{\hat{H}}+\dot{\hat{B}}.\hat{H}\right)-\frac{1}{\hat{\theta }}\hat{q}.\hat{\nabla }\theta \geq 0.$$

The Helmholtz free energy is assumed as a locally defined function and can be characterized by observable variables such as temperature and strain and other non-observable variables that characterize internal rearrangements of a material’s microstructure such as isotropic hardening and kinematic hardening (*cf*. [97,104]). In this model, the Helmholtz free energy is assumed to be a function of the following independent state variables: the product of elastic strain and damage stretch $E_{e}C_{\varphi }$, temperature θ, magnetic field flux density *B*, and a set of *i* number of strain-like internal variables ISVs ${\hat{\Pi }}_{i}$ that are given as follows:

(46) $$\psi =\hat{\psi }\left(E_{e}C_{\varphi },B,\theta ,{\hat{\Pi }}_{i}\right).$$

The ISVs (${\hat{\Pi }}_{i}$), are chosen to represent irreversible mechanisms related to the internal rearrangement of the material microstructure caused by externally applied magnetic, thermal, and mechanical fields. The evolution of ISVs induces strain fields within the domains and changes the electron spin motion on an electronic scale. The ISVs of this model are given as follows:

(47) $${\hat{\Pi }}_{i}=\beta C_{\varphi },\varepsilon_{s}t_{\varphi },\hat{M}$$

where β is the strain-like quantity due to the kinematic hardening describing the effects of geometrically necessary dislocation density (GND) evolution, $\varepsilon_{s}$ is the strain-like quantity due to the isotropic hardening describing the statistically stored dislocation density (SSD) effects, and $\hat{M}$ is the total magnetization of the material. Magnetization nonlinearity occurs due to the rotation and the growth of the magnetic domains. Magnetization refers to which the material can be magnetized when subjected to an external magnetic field. Therefore, the Helmholtz free energy function in Equation (47) may be expressed as

(48) $$\psi =\hat{\psi }(E_{e}C_{\varphi },B,\theta ,\hat{\beta }{\hat{C}}_{\varphi },{\hat{\varepsilon }}_{s}{\hat{t}}_{\varphi },\hat{M}).$$

Assuming that the Helmholtz free energy can be additively decomposed,

(49) $$\begin{array}{l} \hat{\psi }=\hat{\psi }(E_{e}C_{\varphi },B,\theta ,\hat{\beta }{\hat{C}}_{\varphi },{\hat{\varepsilon }}_{s}{\hat{t}}_{\varphi },\hat{M})\\ ={\hat{\psi }}_{E_{e}C_{\varphi }}\left({\hat{E}}_{e}{\hat{C}}_{\varphi },\theta \right)+{\hat{\psi }}_{\hat{B}}\left(\hat{B},\theta \right)+{\hat{\psi }}_{\beta C_{\varphi }}\left(\hat{\beta }{\hat{C}}_{\varphi },\theta \right)+{\hat{\psi }}_{\varepsilon_{s}t_{\varphi }}\left({\hat{\varepsilon }}_{s}{\hat{t}}_{\varphi },\theta \right)+{\hat{\psi }}_{\hat{M}}\left(\hat{M},\theta \right). \end{array}$$

Given these ISVs, the time rate of change in Helmholtz free energy is derived as

(50) $$\dot{\hat{\psi }}=\frac{\partial \hat{\psi }}{\partial \left({\hat{E}}_{e}{\hat{C}}_{\varphi }\right)}:{\dot{\hat{E}}}_{e}{\hat{C}}_{\varphi }+\frac{\partial \hat{\psi }}{\partial \left({\hat{E}}_{e}{\hat{C}}_{\varphi }\right)}:{\hat{E}}_{e}{\dot{\hat{C}}}_{\varphi }+\frac{\partial \hat{\psi }}{\partial \left(\hat{B}\right)}\dot{\hat{B}}+\frac{\partial \hat{\psi }}{\partial \left({\hat{\varepsilon }}_{s}{\hat{t}}_{\varphi }\right)}{\dot{\hat{\varepsilon }}}_{s}{\hat{t}}_{\varphi }+\frac{\partial \hat{\psi }}{\partial \left({\hat{\varepsilon }}_{s}{\hat{t}}_{\varphi }\right)}{\hat{\varepsilon }}_{s}{\dot{\hat{t}}}_{\varphi }+\frac{\partial \hat{\psi }}{\partial \left(\hat{\beta }{\hat{C}}_{\varphi }\right)}\dot{\hat{\beta }}{\hat{C}}_{\varphi }+\frac{\partial \hat{\psi }}{\partial \left(\hat{\beta }{\hat{C}}_{\varphi }\right)}\hat{\beta }{\dot{\hat{C}}}_{\varphi }+\frac{\partial \hat{\psi }}{\partial (\theta )}\dot{\theta }+\frac{\partial \hat{\psi }}{\partial \left(\hat{M}\right)}\dot{\hat{M}}.$$

The setting of the thermodynamic conjugates corresponding to the aforementioned magnetism internal state variables is as follows:

(51) $$\hat{y}=\frac{\partial \hat{\psi }}{\partial \left(\hat{M}\right)}.$$

Substituting the free energy rate (Equation (50)) and (Equation (51)) into the C-D Inequality (Equation (42)) yields

(52) $$-\hat{\rho }(\frac{\partial \hat{\psi }}{\partial \left({\hat{E}}_{e}{\hat{C}}_{\varphi }\right)}:{\dot{\hat{E}}}_{e}{\hat{C}}_{\varphi }+\frac{\partial \hat{\psi }}{\partial \left(\hat{B}\right)}\dot{\hat{B}}+\frac{\partial \hat{\psi }}{\partial \left({\hat{E}}_{e}{\hat{C}}_{\varphi }\right)}:{\hat{E}}_{e}{\dot{\hat{C}}}_{\varphi }+\frac{\partial \hat{\psi }}{\partial \left({\hat{\varepsilon }}_{s}{\hat{t}}_{\varphi }\right)}{\dot{\hat{\varepsilon }}}_{s}{\hat{t}}_{\varphi }+\frac{\partial \hat{\psi }}{\partial \left({\hat{\varepsilon }}_{s}{\hat{t}}_{\varphi }\right)}{\hat{\varepsilon }}_{s}{\dot{\hat{t}}}_{\varphi }+\frac{\partial \hat{\psi }}{\partial \left(\hat{\beta }{\hat{C}}_{\varphi }\right)}\dot{\hat{\beta }}{\hat{C}}_{\varphi }+\frac{\partial \hat{\psi }}{\partial \left(\hat{\beta }{\hat{C}}_{\varphi }\right)}\hat{\beta }{\dot{\hat{C}}}_{\varphi }+\frac{\partial \hat{\psi }}{\partial \left(\hat{\theta }\right)}\dot{\theta }+\;\hat{y}.\dot{\hat{M}})-\hat{\rho }\dot{\hat{\theta }}\hat{s}+\hat{S}:\dot{\hat{E}}+\left(\hat{B}.\dot{\hat{H}}+\dot{\hat{B}}.\hat{H}\right)-\frac{1}{\hat{\theta }}\hat{q}.\hat{\nabla }\hat{\theta }\geq 0.$$

Based on the model developed by Bammann and Solanki (2010) [97], an increasingly strong interaction between some individual dislocation strain fields and their neighboring dislocations induces more dislocation motion that causes material hardening. Therefore, the thermodynamic conjugates, which are stress-like quantities, of the ISVs associated with the stored dislocation and geometrically necessary densities are $\hat{\kappa }$ and $\hat{\alpha }$, and are given as follows:

(53) $$\hat{\kappa }=\hat{\rho }\frac{\partial \hat{\psi }}{\partial \left({\hat{\varepsilon }}_{s}{\hat{t}}_{\varphi }\right)}{\hat{t}}_{\varphi },\;\hat{\alpha }=\hat{\rho }\frac{\partial \hat{\psi }}{\partial \left(\hat{\beta }{\hat{C}}_{\varphi }\right)}{{\hat{C}}_{\varphi }}^{T}.$$

Substituting Equation (50) into Equation (51) yields

(54) $$\begin{array}{l} \left(-\hat{\rho }\frac{\partial \hat{\psi }}{\partial \left({\hat{E}}_{e}{\hat{C}}_{\varphi }\right)}:{\hat{U}}_{\varphi }+\hat{S}\right):{\dot{\hat{E}}}_{e}+\left(-\hat{\rho }\frac{\partial \hat{\psi }}{\partial \left(\hat{\theta }\right)}-\hat{\rho }\hat{s}+\zeta \left(\theta \right)\underline{I}\right)\dot{\theta }+(\frac{1}{2}\hat{S}-\rho \frac{\partial \hat{\psi }}{\partial \left(\hat{\beta }{\hat{C}}_{\varphi }\right)}\hat{\beta }-\\ \frac{1}{3}I\rho \frac{\partial \hat{\psi }}{\partial \left({\hat{\varepsilon }}_{s}{\hat{t}}_{\varphi }\right)}{\hat{\varepsilon }}_{s}-\hat{\rho }\frac{\partial \hat{\psi }}{\partial \left({\hat{E}}_{e}{\hat{C}}_{\varphi }\right)}{\hat{E}}_{e}){\dot{\hat{C}}}_{\varphi }+\left(\hat{H}-\hat{\rho }\frac{\partial \hat{\psi }}{\partial \left(\hat{B}\right)}\right)\dot{\hat{B}}+\left(\hat{B}.\dot{\hat{H}}\right)+\hat{S}:{\dot{\hat{E}}}_{H}-\hat{\rho }\hat{y}.\dot{\hat{M}}-\hat{\kappa }{\dot{\varepsilon }}_{s}-\\ \hat{\alpha }\dot{\beta }+\hat{S}:{\dot{\hat{E}}}_{p}-\frac{1}{\hat{\theta }}\hat{q}.\hat{\nabla }\hat{\theta }\geq 0, \end{array}$$

where

(55) $$\dot{\hat{E}}={\dot{\hat{E}}}_{e}+{\dot{\hat{E}}}_{\theta }+{\dot{\hat{E}}}_{\varphi }+{\dot{\hat{E}}}_{H}+{\dot{\hat{E}}}_{p}.$$

In Equation (54), the damage and the thermal strain rates are given by Dimitrov et al. (2019) as

(56) $${\dot{\hat{E}}}_{\varphi }=\frac{1}{2}{\dot{\hat{C}}}_{\varphi },\;and\;{\dot{\hat{E}}}_{\theta }=\varrho \left(\theta \right)\underline{I}\dot{\theta }.$$

Unlike the other listed strains, the thermal expansion strain is considered a nonlocal variable in this study. We assume the thermal expansion is adequately represented by the linear coefficient of thermal expansion (ϱ) and the temperature increment (Δθ), as previously presented by Dimitrov et al. (2019) [105]:

(57) $${\hat{E}}_{\theta }={\hat{E}}_{\theta }\left(\theta \right)=\frac{1}{2}\left({\hat{C}}_{\theta }-I\right)=\frac{1}{2}\left[2\varrho \Delta \theta I+{\left(\varrho \Delta \theta \right)}^{2}I\right].$$

For most practical applications, the coefficient of thermal expansion exhibits minimal temperature dependence and is considered constant within a small temperature range, below the Curie temperature for magnetic materials [106]. The material time derivative of the thermal expansion strain (${\dot{\hat{E}}}_{\theta }$) in the local form is then approximated as follows:

(58) $${\dot{\hat{E}}}_{\theta }=\frac{\partial }{\partial \theta }{\hat{E}}_{\theta }\dot{\theta }=\varrho \left(\theta \right)I\dot{\theta }+\frac{\partial \varrho }{\partial \theta }I\dot{\theta }\approx \varrho \left(\theta \right)I\dot{\theta }.$$

Based on the scheme used by Coleman and Gurtin (1967) [55] and Kratochvil and Dillon (1969) [107], the constitutive equations for stress, entropy, and magnetism for this continuum model are given as follows:

(59) $$\begin{array}{l} \hat{S}=\hat{\rho }\frac{\partial \hat{\psi }}{\partial \left({\hat{E}}_{e}{\hat{C}}_{\varphi }\right)}:{\hat{C}}_{\varphi },\\ \hat{s}=-\frac{\partial \hat{\psi }}{\partial \left(\theta \right)}+\frac{1}{\hat{\rho }}\tilde{\varrho }tr\left(\underline{\tilde{S}}\right),\\ \left(\frac{1}{2}\hat{S}-\rho \frac{\partial \hat{\psi }}{\partial \left(\hat{\beta }{\hat{C}}_{\varphi }\right)}\hat{\beta }-\frac{1}{3}I\rho \frac{\partial \hat{\psi }}{\partial \left({\hat{\varepsilon }}_{s}{\hat{t}}_{\varphi }\right)}{\hat{\varepsilon }}_{s}-\hat{\rho }\frac{\partial \hat{\psi }}{\partial \left({\hat{E}}_{e}{\hat{C}}_{\varphi }\right)}{\hat{E}}_{e}\right){\dot{\hat{C}}}_{\varphi }=0,\\ -\hat{\rho }\frac{\partial \hat{\psi }}{\partial \left(\hat{B}\right)}+\hat{H}=0, \end{array}$$

where ***H***, S and ***s*** are considered as thermodynamic forces associated with ***B***, $E_{e},\;\mathrm{and}\;\theta ,$ respectively.

Using Equation (59), the dissipation energy inequality (Equation (54)) can be reduced to

(60) $$\left(\hat{B}.\dot{\hat{H}}\right)+\hat{S}:{\dot{\hat{E}}}_{H}-\hat{\rho }\hat{y}.\dot{\hat{M}}-\hat{\kappa }{\dot{\varepsilon }}_{s}-\hat{\alpha }:\dot{\beta }+\hat{S}:{\dot{\hat{E}}}_{p}-\frac{1}{\theta }\hat{q}.\hat{\nabla }\theta \geq 0.$$

Following the classical definition of entropy and neglecting second-order effects, Equation (40) can be approximated as part of the internal energy that dissipates as specific heat. Equation (41) is assumed to equal the portion of the internal energy that is stored as reversible processes or converted to irreversible damage and dislocation structure evolution. Consequently, from the definition of specific heat per unit mass ($c_{M}=du/d\theta$), we write the temperature evolution equation:

(61) $$\dot{\theta }=\frac{1}{\tilde{\rho }{\tilde{c}}_{M}}\left\{\tilde{S}:{\dot{\tilde{E}}}_{p}-{\tilde{\kappa }}_{s}{\dot{\tilde{\varepsilon }}}_{s}-\tilde{\alpha }:\dot{\tilde{\beta }}-\tilde{\nabla }.\tilde{q}+\tilde{\rho }\tilde{r}+\hat{\rho }\frac{\partial \hat{\psi }}{\partial \left(\hat{B}\right)}\dot{\hat{B}}+\left(\dot{\hat{H}}.\hat{B}+\hat{H}.\dot{\hat{B}}\right)+\hat{S}:{\dot{\hat{E}}}_{H}-\hat{\rho }\hat{a}.\dot{\hat{M}}\right\}.$$

## 5. Kinetics

### 5.1. Experimental Magnetostriction Test

**Figure 5:**
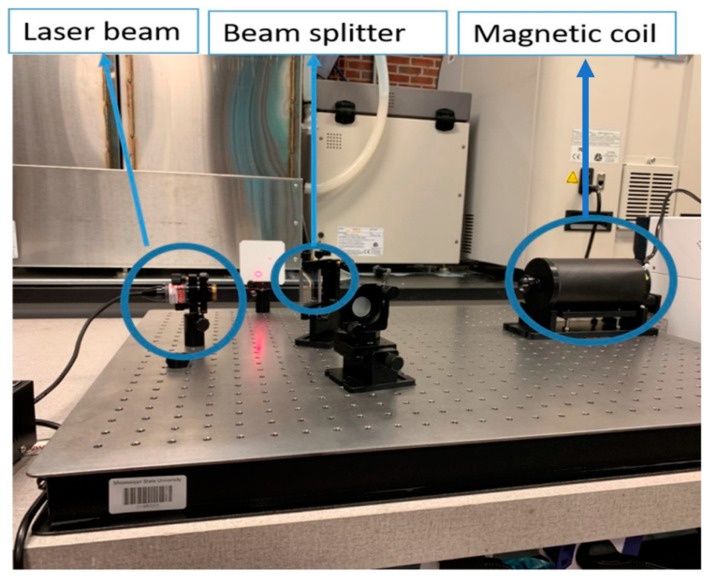
The Michelson Interferometer setup used to measure the magnetostriction in this study. The iron (Fe), nickel (Ni), and cobalt (Co) specimens were placed in the magnetic coil.

**Figure 6:**
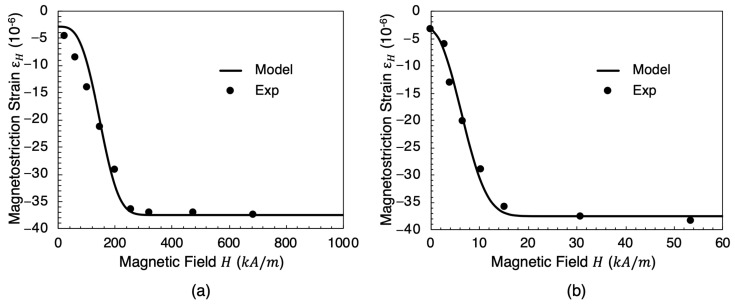
Magnetostriction variation ($\varepsilon^{H}$) with respect to the external magnetic field *H* (kA/m) for soft magnets: (**a**) cobalt (Co) and (**b**) nickel (Ni). Symbols are experimental data obtained in the part of the present study and lines are for the model.

**Figure 7:**
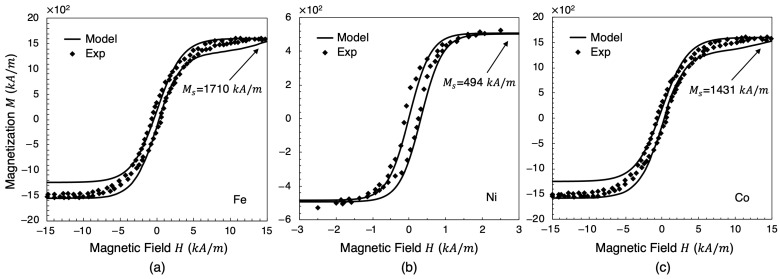
Magnetization variation *M* (kA/m) with respect to the external magnetic field *H* (kA/m), for soft magnets: (**a**) iron (Fe), (**b**) nickel (Ni), and (**c**) cobalt (Co). Symbols are experimental data [108] and lines are for the model.

In order to examine the effects of mechanical stress and magnetic field, experiments were conducted on the three rod specimens (length of 185 mm and diameter of 6 mm) of iron, nickel, and cobalt. The specimen dimension is appropriate to place them inside of the magnetic coils. The experiments quantified the magnetostriction of the rod specimens. The apparatus used in this experiment is the Michelson Interferometer. The Michelson Interferometer is an optical method used to measure the magnetostriction. The Michelson Interferometer emits a laser wave that is then divided into two parts. Each of the new light beams travels a different path that recombines together. The magnetostriction strain is equal to the small mirror movement once the sample is subjected to a magnetic field and starts to elongate. Figure 5 shows the Michelson Interferometer used in this study. We applied various intensities of external magnetic fields (various electrical currents), and the obtained relationship among external magnetic field, magnetostriction, and magnetization is presented in Figure 6 and Figure 7, respectively. 

### 5.2. Cauchy Stress Tensor

The frame indifferent Jaumann rate of the Cauchy stress was initially developed by Bammann (1990) [99] as a function of kinematics and elastic properties. It was then extended by Horstemeyer and Gokhale (1999) [109] to capture the degradation of a material’s effective stiffness by damage. Therefore, the frame indifferent elastic stress rate in the current configuration (R) is given as follows:

(62) $$\overset{○}{\sigma }=\dot{\sigma }\text{-}W_{e}\sigma +\sigma {W_{e}}^{T}=\dot{\sigma }\text{-}W_{e}\sigma +\sigma W_{e}=\lambda \left(1-\varphi \right)tr\left(D_{e}\right)I+2\mu \left(1-\varphi \right)D_{e}-\frac{\dot{\varphi }}{\left(1-\varphi \right)}\sigma ,$$

where λ, μ are the Lamé constants, φ is the total damage, $D_{e}$ is the elastic rate deformation, and $W_{e}$ is the elastic spin which is given as follows:

(63) $$W_{e}=W-W_{H}-W_{p},$$

where $W_{p}$ is the plastic spin and $W_{H}$ is the magnetic spin. The magnetic spin term is nonzero because of the electron spin motion distribution of the electrons within the atoms.

The elastic rate of deformation $D_{e}$, is given as the difference between the total rate of deformation and the plastic, magnetic, damage, and thermal rates of deformation ($D_{p}$,$\;D_{H},D_{\varphi },\;\mathrm{and}\;D_{\theta }$):

(64) $$D_{e}=D-D_{p}-D_{H}-D_{\varphi }-D_{\theta }.$$

The plastic deformation rate is given using the strain flow rule, which was initially developed by Bammann (1990) [99] in order to relate the deviatoric rate of deformation to the applied stress and ISVs, then extended by Horstemeyer and Gokhale (1999) [109]. The plastic strain flow rule is the tensor rate at which the distances between a point (*P*) and its neighboring particles deform plastically, and it is given as follows:

(65) $$D_{p}=\sqrt{\frac{3}{2}}f\left(\theta \right)\times \sin h\left[\frac{\sqrt{\frac{3}{2}}\|\sigma^{\prime }-\sqrt{\frac{2}{3}}\alpha \|-\left\{R+Y\left(\theta \right)\right\}\left\{1-\varphi \right\}}{V\left(\theta \right)\left\{1-\varphi \right\}}\right]\times \frac{\sigma^{\prime }-\sqrt{\frac{2}{3}}\alpha }{\left\|\sigma^{\prime }-\sqrt{\frac{2}{3}}\alpha \right\|}.$$

The thermal and damage deformation rate were developed in a similar way by Bammann (1990) [99] and Horstemeyer et al. (2000) [110], respectively, and given as follows:

(66) $$D_{\theta }=\alpha_{th}\dot{\theta }I,$$

(67) $$D_{\varphi }=\frac{\varphi }{3\left(1-\varphi \right)}I.$$

The magnetic deformation rate is derived in this work as follows:

(68) $$D_{H}=\pm c\left(-bp\exp\left(\frac{{\left(H⊗H^{T}+\chi \right)}^{p}}{\|H⊗H^{T}+\chi {\|}^{q}}\right)*\frac{{\left(H⊗H^{T}+\chi \right)}^{p-1}}{\|H⊗H^{T}+\chi {\|}^{q}}*\dot{H}\right)*\frac{H⊗H^{T}+\chi }{\left\|H⊗H^{T}+\chi \right\|}+D_{H}^{MX},$$

For the sake of simplicity, we set the magnetic field vector and its transpose dyadic product as follows:

(69) $$\Gamma =H⊗H^{T}.$$

Therefore, the magnetic deformation rate is written as

(70) $$D_{H}=\pm c\left(-bp\exp\left(\frac{{\left(\Gamma +\chi \right)}^{p}}{\|\Gamma +\chi {\|}^{q}}\right)*\frac{{\left(\Gamma +\chi \right)}^{p-1}}{\|\Gamma +\chi {\|}^{q}}*\dot{H}\right)*\frac{\Gamma +\chi }{\left\|\Gamma +\chi \right\|}+D_{H}^{MX}.$$

In this case, the Maxwell-associated deformation rate ($D_{H}^{MX}$) is assumed to be zero, since the deformation caused by the Maxwell stress is zero; thus, the magnetic deformation rate is written as follows:

(71) $$D_{H}=\pm c\left(-bp\exp\left(\frac{{\left(\Gamma +\chi \right)}^{p}}{\|\Gamma +\chi {\|}^{q}}\right)*\frac{{\left(\Gamma +\chi \right)}^{p-1}}{\|\Gamma +\chi {\|}^{q}}*\dot{H}\right)*\frac{\Gamma +\chi }{\left\|\Gamma +\chi \right\|}.$$

Functions $f\left(\theta \right)$,$\;Y\left(\theta \right),$ and $V\left(\theta \right)$ are functions that have an Arrhenius-type temperature dependence. They were developed by Bammann (1990) [99] and are given as follows:

(72) fθ=C5exp−C6θ,Yθ=C3expC4θ,Vθ=C1exp−C2θ,

where *Y(*θ*)* is the rate-independent yield stress. Function $f\left(\theta \right)$ determines when the rate dependences affect initial yielding. Function $V\left(\theta \right)$ determines the magnitude of the rate dependence on yielding. These functions are easily determined from simple isothermal compression tests with different strain rates and temperatures. $C_{1}$, $C_{2}$,$\;C_{3}$,$\;C_{4}$, $C_{5}$, and $C_{6}$ are Arrhenius-type temperature-dependent calibration constants.

Kinematic hardening internal state variable α represents the anisotropic effect of the dislocation density while isotropic hardening internal state variable *R* mimics the global dislocation density effect. The kinematic hardening rate equation was developed by Bammann (1990) [99] and then extended by Tucker et al. (2010) [111] to account for the grain size effect,

(73) $$\overset{○}{\alpha }=\dot{\alpha }-W_{e}\alpha +\alpha W_{e}=h\left(\theta \right)D_{p}-\left[\sqrt{\frac{2}{3}}r_{d}\left(\theta \right)\|D_{p}\|+r_{s}\left(\theta \right)\right]\sqrt{\frac{2}{3}}\|\alpha \|\alpha ){\left(\;\frac{DCS0}{DCS}\right)}^{Z},$$

where

(74) rdθ=C71−C19427−J32J23−C20J3J21.5exp−C8θ,hθ=C91+C19427−J32J23+C20J3J21.5exp−C10θ,rsθ=C11exp−C12θ.

The isotropic hardening rate equation is prescribed in a hardening minus recovery format which accounts for the grain size effect and is presented by Tucker et al. (2010) [111] as follows:

(75) $$\dot{\kappa }=\sqrt{\frac{2}{3}}H\left(\theta \right)D_{p}-\left[\sqrt{\frac{2}{3}}R_{d}\left(\theta \right)\|D_{p}\|+R_{s}\left(\theta \right)\right]\kappa^{2}{\left(\;\frac{DCS0}{DCS}\right)}^{Z},$$

where κ is the isotropic hardening, *H* is the work hardening modulus, $R_{d}\left(\theta \right)$ is the dynamic recovery that captures the dislocation glide effect, $R_{s}\left(\theta \right)$ is the static recovery that captures the dislocation climb or the diffusion effect, and $D_{in}=\sqrt{\frac{2}{3}}{\dot{\varepsilon }}^{p}N$ is the deviatoric inelastic strain rate. *DCS0* and *DCS* represent the initial average grain size and the average grain size that directly influence the dislocation density and thereby interact with the hardening parameters, respectively. *Z* is a constant exponent for the grain size effect on hardening. 

The parameters of these mechanisms are given [72] as follows:

(76) Rdθ=C131−C19427−J32J23−C20J3J21.5exp−C14θ,Hθ=C151+C19427−J32J23+C20J3J21.5exp−C16θ,Rsθ=C17exp−C18θ,

where $J_{2}$ and $J_{3}$ are the second and third invariants of deviatoric stress, respectively.

The equations describing the material’s degradation (or total damage) were developed by Horstemeyer et al. (2000) [72] (void volume fraction) based on the consideration of the microphysical damage mechanism. They are given as follows:

(77) ϕ=ηvc,

where η, v, and c represent void nucleation, growth, and coalescence, respectively. The total damage rate of the void volume fraction within a ductile metal is given as follows:

(78) $$\dot{\phi }=\dot{\eta }vc+\eta \dot{v}c+\eta v\dot{c}.$$

The rate evolution of the void nucleation/growth and coalescence were described independently by Horstemeyer et al. (2000) [72]. The void nucleation rate is given as follows:

(79) $$\dot{\eta }=\frac{d^{\frac{1}{2}}}{K_{ic}f^{\frac{1}{3}}}\eta \left(a\left[\frac{4}{27}-\frac{J_{3}^{2}}{J_{2}^{3}}\right]+b\frac{J_{3}}{J_{2}^{\frac{3}{2}}}+c\|\frac{I_{1}}{\sqrt{J_{2}}}\|\right)\|D_{d}\|\exp\left(\frac{C_{\eta T}}{T}\right),$$

where d and f are material property constants of the initial secondary phase particle size and volume fraction, respectively. $I_{1}$, $J_{2}$, and $J_{3}$ are the first, the second, and third stress invariants representing the stress dependence of the void nucleation rate. Calibration constants a, b, and c represent the material’s torsional for void nucleation, the difference between the tension and compression, and the stress triaxiality sensitivity for void nucleation, respectively, and they are all determined experimentally (based on tension, compression, and torsion tests at different strain levels). $C_{\eta T}$ is the calibration constant used to control thermal sensitivity during the void nucleation phase.

Void nucleation for a bar subjected to uniaxial stress for which the deformation is isothermal and happens at a constant strain rate can be obtained by an integration of Equation (79). This is given, as follows, by Bammann (1990) [99]:

(80) $$\eta =\eta_{0}\exp\left[\|E\|\frac{d^{\frac{1}{2}}}{K_{ic}f^{\frac{1}{3}}}\eta \left(a\left[\frac{4}{27}-\frac{J_{3}^{2}}{J_{2}^{3}}\right]+b\frac{J_{3}}{J_{2}^{\frac{3}{2}}}+c\|\frac{I_{1}}{\sqrt{J_{2}}}\|\right)\exp\left(\frac{C_{\eta T}}{T}\right)\right],$$

where ‖E‖ is the norm of the total Lagrangian strain tensor.

The rate evolution of the second phase particle growth was developed by McClintock (1964) [112]. Among all damage models, McClintock’s model is the most used one since it can be used at different strain/hardening rates, different temperatures, and different stress triaxialities. The void growth rate is therefore given as follows:

(81) $$\dot{v}=\frac{4\pi }{3}{\left(\frac{\sqrt{3}d_{v0}}{4\left(1-n\right)}\sin h\left[\sqrt{3}\left(1-n\right)\frac{\sqrt{2}I_{1}}{3\sqrt{J_{2}}}\right]\|D_{d}\|\right)}^{3}$$

such that $d_{v0}$ is the initial void diameter and *n* is the McClintock growth rate constant originally motivated by the material hardening rate.

The void growth equation for an increasing strain and/or stress triaxiality is given by McClintock (1964) [112] as follows:

(82) $$v=\frac{4\pi }{3}{\left(d_{v0}\exp\left[\|E\|\frac{\sqrt{3}}{2\left(1-n\right)}\sin h\left(\sqrt{3}\left(1-n\right)\frac{\sqrt{2}I_{1}}{3\sqrt{J_{2}}}\right)\right]\right)}^{3}.$$

As the applied stress increases, and as the voids nucleate within the material, voids tend to coalesce, resulting in a void sheet or a natural void. The coalescence rate evolution is described by Tucker et al. (2010) [111] and is given as follows:

(83) $$\dot{c}=\left[c_{d1}+c_{d2}\left(\eta \dot{v}+\dot{\eta }v\right)\right]\exp^{C_{CT}T}{\left(\frac{DCS0}{DCS}\right)}^{z},$$

where $c_{d1}$ and $c_{d2}$ are calibration constants, and DCS0 and DCS represent the initial average grain size and the average grain size that directly influence the dislocation density and as such interact with the hardening parameters, respectively. *Z* is a constant exponent for the grain size effect on hardening. $C_{CT}$ is a thermal sensitivity calibration constant for void coalescence. 

The co-rotational Jaumann rate is given as follows:

(84) $$\overset{○}{\sigma }=Y\dot{\varepsilon^{e}}\left(1-\varphi \right)+Y\varepsilon^{e}\left(1-\dot{\varphi }\right)+\dot{Y}\varepsilon^{e}\left(1-\varphi \right)-W_{e}\sigma +\sigma W_{e},$$

(85) $$\overset{○}{\sigma }=Y\dot{\varepsilon^{e}}\left(1-\varphi \right)+Y\varepsilon^{e}\left(1-\dot{\varphi }\right)+\dot{Y}\varepsilon^{e}\left(1-\varphi \right)-W_{e}[(Y^{M}\varepsilon^{e}+Y^{M}\varepsilon^{t}+Y^{M}\varepsilon^{\varphi }+Y^{M}\varepsilon^{MS}\;+Y^{M}\varepsilon^{MX})\left(1-\varphi \right)]+\sigma W_{e}$$

where the elastic strain rate is given as

(86) $${\dot{\varepsilon }}^{e}=\dot{\varepsilon }-{\dot{\varepsilon }}^{p}-{\dot{\varepsilon }}^{\varphi }-{\dot{\varepsilon }}^{\theta }-{\dot{\varepsilon }}^{H}.$$

Assuming isotropic damage-induced deformation, the damage-induced strain (the volumetric strain related to the nucleation, growth, and coalescence of voids) is given by Horstemeyer et al. (1999) [109] as follows:

(87) $${\dot{\varepsilon }}^{\varphi }=\frac{1}{3}{\left(1-\varphi \right)}^{-1}\dot{\varphi }I,$$

which illustrates the damage-related strain rate change with respect to the damage parameter, in this case related to the nucleation, growth, and coalescence of the voids within the material.

The strain arising from thermal expansion and contraction is given by Francis et al. (2014) [71] as follows:

(88) $${\dot{\varepsilon }}^{\theta }=\alpha_{th}\Delta \theta .$$

Equation (72) shows the main relationship between the magnetostriction strain and the magnetic field for ferromagnetic material.

(89) $$\varepsilon^{MS}=\pm \left(1-\exp\left(\frac{{\left(\Gamma +a\right)}^{p}}{\|\Gamma +a{\|}^{q}}\right)*b\right)*c\pm d,$$

where a is the normal component χ, c,d,p, and q are calibration constants, b is the magnetostriction constant at the saturation level, and *H* is the external magnetic field known to vary with respect to time. The magnetostriction strain rate is given as follows:

(90) $${\dot{\varepsilon }}^{MS}=\pm c\left(-bp\exp\left(\frac{{\left(\Gamma +a\right)}^{p}}{\|\Gamma +a{\|}^{q}}\right)*\frac{{\left(\Gamma +a\right)}^{p-1}}{\|\Gamma +a{\|}^{q}}*\dot{H}\right).$$

The magnetostriction strain equation is compared with experimental data obtained from the Michelson interferometer for the two ferromagnetic materials: nickel (Ni) and cobalt (Co), as shown in Figure 6. Since the magnetic flux density strain is so small, it is assumed that $\varepsilon^{MX}$ takes a constant value that depends on the magnetic field applied in the material. Therefore, the magnetic flux density strain rate (${\dot{\varepsilon }}^{MX}$) is assumed to be equal to zero.

(91) $${\dot{\varepsilon }}^{MX}=0.$$

### 5.3. An Internal State Variable for Magnetization

To capture the dissipative and the hysteretic response of magnetostrictive materials, the use of internal state variables is necessary. Magnetization is defined as the material’s response to an external magnetic field (*H*). It is the average of the magnetic domains’ moments. Paramagnetic and diamagnetic materials have no magnetization (or if they do it is a negligible one), unless it is subjected to a magnetic field. Once the magnetic field is removed, the material loses its magnetization. Ferromagnetic, ferrimagnetic, and antiferromagnetic materials all have magnetization even when no magnetic field (*H*) is applied. When an external magnetic field (*H*) is applied, the ferromagnetic, ferrimagnetic, and antiferromagnetic materials exhibit a nonlinear magnetization with respect to the magnetic field (*H*), as shown in Figure 7.

Previous models were developed to describe the hysteresis behavior of magnetic material. The most known model is the Jiles–Atherton Model [14,46,113] which describes the magnetization (*M*) behavior with respect to the magnetic field (*H*) through an ordinary differential equation. Differential equations require significant computational resources.

In this work, magnetization is assumed to be one of the internal state variables describing the magnetic domain behavior when subjected to magnetic field (*H*). Based on the hysteresis behavior, the magnetization rate evolution is written in a simpler form than previous models, which allows for simple numerical implementation and is given as follows:

(92) $$\dot{M}\left(H\right)=\dot{\xi }\left(H\right)+\dot{\chi }\left(H\right),$$

such that $\dot{\xi }\left(H\right)$ is the isotropic magnetization rate (needs identity matrix) and $\dot{\chi }\left(H\right)$ is the anisotropic magnetization rate given as follows:

(93) $$\dot{\xi }\left(H\right)=\dot{H}*\left(\frac{M_{s}}{A}{*\sec h}^{2}\left(\frac{H}{A}\right)-\frac{1}{B}\exp\left(-\frac{H}{B}\right)\right)\;\dot{\chi }\left(H\right)=\dot{\Gamma }*\left(\frac{M_{s}}{Q}{*\sec h}^{2}\left(\frac{\Gamma }{P}\right)+\frac{R}{P}\left(\sin h\left(\frac{\Gamma }{Q}\right)\right)\right),$$

where *A*, *B*, *P*, *Q*, and *R* are constants of the material, $M_{s}$ is the saturation magnetization. 

These equations are compared with available experimental data [108] for magnetization of iron, nickel, and cobalt at various magnetic field strengths as shown in Figure 7. The results show an acceptable approximation to the experimental results.

## 6. Discussion

In this paper, we developed an Internal State Variable (ISV) constitutive model to account for magnetism-dependent elasto-viscoplastic and damage model for magnetic materials that brings in three novel ideas: (1) it is new to analyze the kinematics of the deforming continuum body under the external magnetic field to account for elastic/inelastic deformation and vorticity affected by the magnetic field; (2) this is the first paper (to the best of our knowledge) that introduces a new magnetic internal state variable constrained by the first and second laws of thermodynamics (Clausius–Duhem inequality); and (3) this ISV-based constitutive modeling is a novel approach, by itself, to this particular problem. The ISV model integrates the effect of a new magnetic observable variable: magnetic flux. Magnetic flux is the main macroscale constraint to which ferromagnetic materials are subjected during operation in most applications. To predict the behavior of ferromagnetic materials, the model included magnetization variations with respect to the magnetic field as an ISV that was then experimentally validated. Even though the strain response due to the magnetic field was small compared to the mechanical and thermal responses, the latter was included as the magnetostriction strain. The magnetostriction strain was experimentally validated for cobalt and nickel. More experiments related to different materials, boundary conditions, and non-monotonic sequences can be explored for future evaluation of the theoretical model.

## 7. Conclusions

In this study, a novel macroscopic constitutive theory is presented to describe the thermal–elastic–plastic damage behavior of magnetic materials. A multiscale and fully coupled multiphysics Internal State Variable (ISV) model is created to describe the effects of magnetic field forces and moments under thermomechanical deformations based on a kinematics, thermodynamics, and kinetics independently developed and subsequently coupled to provide an internally consistent theory for magnetic influenced deformation.

The major contributions are related to developing a model that captures the magnetic effects on deformation using a thermodynamically consistent framework developed by Coleman and Gurtin (1967) [55]. The ISV model features a kinematics description of the deformation using a multiplicative decomposition of the total deformation gradient into elastic, thermal, magnetostrictive, damage, and plastic components. Thermodynamic restrictions are employed using the Clausius–Duhem Inequality which combines the first and second laws of thermodynamics. The kinetic framework enables the prediction of magnetically influenced stresses and strains in materials exposed to magnetic fields. The novel ISV model framework couples elastic, thermal, damage, and plastic effects to magnetic effects. 

To describe the mechanical deformation resulting from the magnetic field, an equation describing the magnetostriction variation with respect to the magnetic field is introduced. The magnetostriction strain is a simple equation, with one variable (magnetic field) and other calibration constants, that can predict the nonlinear behavior of soft and hard magnets. To describe the magnetic behavior of the magnet, magnetization is introduced as an internal state variable for which an equation is developed.

The developed magnetism-dependent ISV constitutive model is compared with experimental data of nickel, cobalt, and iron. From the experiments, we measure the mechanical deformation (magnetostriction strain) of nickel and cobalt and magnetization of iron, nickel, and cobalt when they are subjected to magnetic fields. The magnetostriction strain and the magnetization equations are developed in the framework of ISV theory and compared with the obtained experimental data, and both show good agreement.

For future considerations, we encourage researchers to conduct experiments in which the applied magnetization levels, temperatures, and applied strains at different strain rates and paths are varied. This ISV model framework should be admissible to address the aforementioned topics for use in design. In addition, we are planning to further the model development with a multiscale analysis of large strain deformation with different levels of magnetization. Finally, we recommend that different alloyed systems be examined in the context of this ISV model as only pure metals are used in this study. 

## Figures and Tables

**Table 1:** Five different types of magnetic materials.

Type	Spin Alignment	Spin Illustrated in Simplified Plot	Examples
Ferromagnets	Electron spins align parallel to one another, resulting in a spontaneous magnetization.	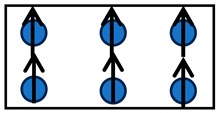	Fe, Co, Ni
Ferrimagnets	Majority of electron’s spins parallel to one another, some spins are antiparallel, resulting in spontaneous magnetization.	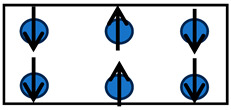	Magnetite (${Fe}_{3}O_{4}$), yttrium iron garnet (YIG)
Antiferromagnets	Electron spins align antiparallel to each other, resulting in a null net magnetization.	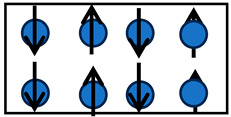	Cr
Paramagnets	Electron spins tend to align parallel when an external magnetic field is applied.	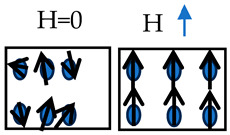	Oxygen, sodium, aluminum, calcium, uranium
Diamagnets	Electron spins tend to align antiparallel to an external magnetic field.	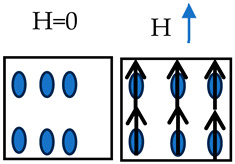	Copper, silver, gold, nitrogen

## Data Availability

The raw data supporting the conclusions of this article will be made available by the authors on request.
